# The Impact and Cost-Effectiveness of a Four-Month Regimen for First-Line Treatment of Active Tuberculosis in South Africa

**DOI:** 10.1371/journal.pone.0145796

**Published:** 2015-12-30

**Authors:** Gwenan M. Knight, Gabriela B. Gomez, Peter J. Dodd, David Dowdy, Alice Zwerling, William A. Wells, Frank Cobelens, Anna Vassall, Richard G. White

**Affiliations:** 1 Centre for Mathematical Modelling of Infectious Diseases, TB Centre, TB Modeling Group, Department of Infectious Disease Epidemiology, London School of Hygiene and Tropical Medicine, London, United Kingdom; 2 Amsterdam Institute for Global Health and Development and Department of Global Health, Academic Medical Center, University of Amsterdam, Amsterdam, The Netherlands; 3 Department of Global Health and Development, London School of Hygiene and Tropical Medicine, London, United Kingdom; 4 School of Health and Related Research, University of Sheffield, Sheffield, United Kingdom; 5 Department of Epidemiology, Johns Hopkins Bloomberg School of Public Health, Baltimore, United States of America; 6 Global Alliance for TB Drug Development, New York, United States of America; 7 KNCV Tuberculosis Foundation, The Hague, The Netherlands; Public Health Agency of Barcelona, SPAIN

## Abstract

**Background:**

A 4-month first-line treatment regimen for tuberculosis disease (TB) is expected to have a direct impact on patient outcomes and societal costs, as well as an indirect impact on *Mycobacterium tuberculosis* transmission. We aimed to estimate this combined impact in a high TB-burden country: South Africa.

**Method:**

An individual based *M*. *tb* transmission model was fitted to the TB burden of South Africa using a standard TB natural history framework. We measured the impact on TB burden from 2015–2035 of introduction of a non-inferior 4-month regimen replacing the standard 6-month regimen as first-line therapy. Impact was measured with respect to three separate baselines (Guidelines, Policy and Current), reflecting differences in adherence to TB and HIV treatment guidelines. Further scenario analyses considered the variation in treatment-related parameters and resistance levels. Impact was measured in terms of differences in TB burden and Disability Adjusted Life Years (DALYs) averted. We also examined the highest cost at which the new regimen would be cost-effective for several willingness-to-pay thresholds.

**Results:**

It was estimated that a 4-month regimen would avert less than 1% of the predicted 6 million person years with TB disease in South Africa between 2015 and 2035. A similarly small impact was seen on deaths and DALYs averted. Despite this small impact, with the health systems and patient cost savings from regimen shortening, the 4-month regimen could be cost-effective at $436 [NA, 5983] (mean [range]) per month at a willingness-to-pay threshold of one GDP per capita ($6,618).

**Conclusion:**

The introduction of a non-inferior 4-month first-line TB regimen into South Africa would have little impact on the TB burden. However, under several scenarios, it is likely that the averted societal costs would make such a regimen cost-effective in South Africa.

## Introduction

South Africa suffers from an extremely high burden of TB, with increasing levels of drug resistance and an ongoing HIV epidemic [1]. It has one of the highest gross domestic products (GDPs) in the WHO-identified 22 high TB burden countries [2]. Recently, the South African Department of Health has invested heavily in TB control with the adoption and rapid rollout of the new diagnostic tool GeneXpert [3]. However, there is still a high level of TB transmission occurring [4] at a large cost to health systems and patients [5–7].

Tackling this burden could be achieved, in part, by using a shortened first-line regimen. The current standard first-line treatment regimen for TB consists of daily administration of four drugs (isoniazid, rifampicin, ethambutol and pyrazinamide, HZRE) for 2 months followed by two drugs (isoniazid and rifampicin) for a further 4 months. Shortening the first-line regimen could reduce the burden on both patient populations and health systems. Several new shortened regimens are in the pipeline utilizing either new combinations of existing drugs or incorporating new compounds that offer promise going forward [8].

Policy makers use a wide range of evidence in order to make decisions. For the adoption of shortened regimens for TB, two such pieces of evidence are likely to be population health impact and cost-effectiveness analysis [9]. These can be assessed using mathematical models that include the impact on patients and disease transmission. Previous mathematical models have investigated the impact of a shortened regimen on the number of TB cases and shown that it is likely to be relatively small (<10%) [10–13]. However, none have estimated the population-level impact and cost-effectiveness in a specific high-burden setting.

In this work we focused on assessing the impact of a hypothetical shortened regimen in a high TB burden country that has a strong track record of investing in TB control, South Africa. By fitting a dynamic transmission model explicitly to the TB epidemic in this country we then assessed the impact of a new shortened regimen of the most likely length (4-months) [8]. We assumed that the regimen was equivalent, being non-inferior and not superior, and thus aimed to explore only the impact of shortening the treatment length. Specifically, we assumed that the number of deaths and proportion of cases cured at treatment completion was the same for both treatments and we did not include any margin of non-inferiority. We expanded previous cost-effectiveness analysis of shortened TB regimens [13] to include new recently collected cost data [6,7] and build upon work presented in a companion paper [14] to conduct a threshold analysis around the cost at which such a regimen could be cost-effective for South Africa, including transmission impact.

## Materials and Methods

We modified a previously published, individual-based simulation model [15,16] to include a shortened TB treatment regimen length and fitted this to the current data on TB and HIV epidemiology in South Africa (see S1 Appendix for full details). We used this to evaluate the difference in TB burden over 2015 and 2035 between a scenario with continued use of the current standard 6-month TB treatment (isoniazid, rifampicin, pyrazinamide, ethambutol: 2HRZE/4RH) vs. a non-inferior shortened 4-month regimen for first-line treatment. We assumed that the new regimen contained rifampicin and thus was ineffective against MDR-TB.

### Natural history of TB

**Fig 1:**
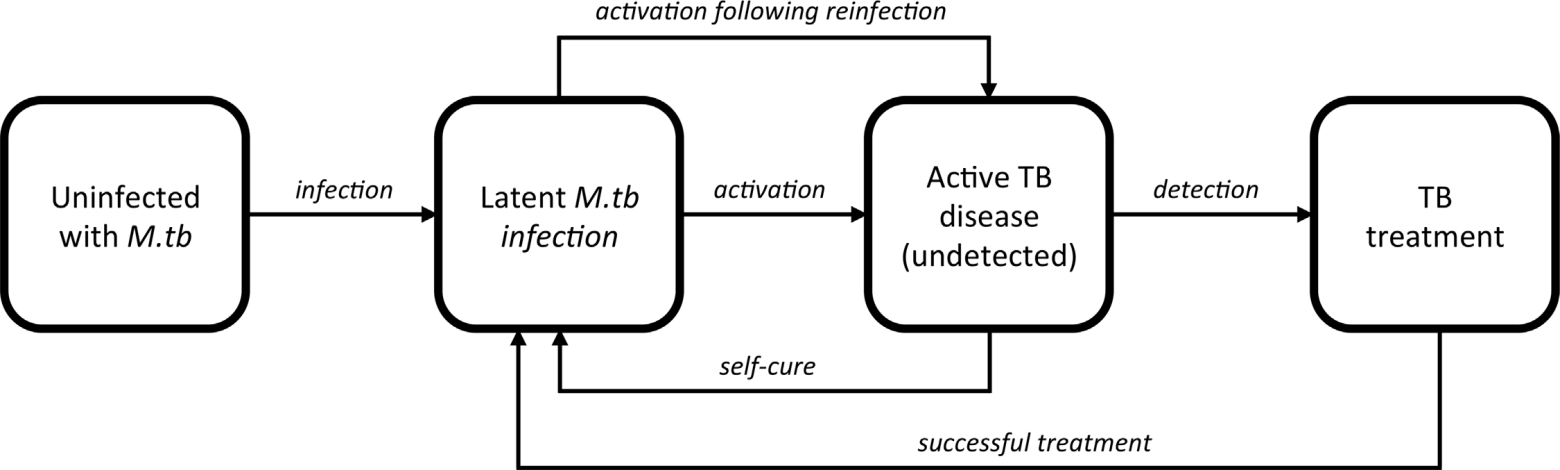
Highly simplified natural history model showing the schematic progression through TB states. These transitions are affected by CD4+ count of those with HIV and by previous TB treatment history status. Age-dependent mixing was also included.

The model has a standard natural history of TB framework (Fig 1) and includes age, HIV status and CD4 count, and TB infection history. Timing of development of TB disease after (re-)infection is influenced by HIV status and CD4 count; HIV status and age influence the probability of TB disease being smear-positive. TB infection from TB cases occurs in the community, the latter with age-dependent mixing. The model is built using C. For more information and all natural history parameters see S1 Appendix.

### Resistance in TB assumptions

The impact of resistance on the probability of transmission is highly uncertain [17]. Therefore, transmission of individual resistance profiles was not monitored in the model, nor was acquired resistance included. Instead we composed our TB cases of a weighted average of proportion of cases with drug sensitive and resistant TB. Two resistance categories were included: pan-susceptible and multi-drug resistant (resistance to isoniazid and rifampicin, MDR-TB) at 2015 levels in the main analysis for treatment-naive patients of 98.2% and 1.8% respectively [2]. These levels were assumed to remain constant from 2015 onwards. We explored this assumption in our scenario analysis (see below). An individual who has received TB treatment before had a higher probability of MDR-TB in subsequent episodes of active disease, determined by a fixed odds ratio (3.72) [18].

### TB Treatment assumptions

First-line therapy is prescribed to all who are detected with active TB. If rifampicin resistance is detected, via an assumed coverage of GeneXpert testing [19] (Table 1), then, dependent on MDR treatment coverage, there is some chance that this patient will be started on 24 month MDR treatment with set outcomes (Tables B&C in S1 Appendix).

**Table 1:** Differences in coverage and adherence to treatment guidelines and other healthcare interventions across the three baselines from 2010. Up to 2010, the Basecase levels were assumed.

		Basecase	Guidelines	Policy	Current
**MDR treatment**	2010 level	17% [18]			
	Scale-up period		2011–2013	2011–2015	2011–2013
	Final level [41]		100%	85% [33]	60% [42]
**GeneXpert**	Percentage detected receiving	23%	100%	100%	90%
	Percentage detected with MDR starting treatment	73%	100%	85%	60.66% [2]
**IPT**	For HIV+ on starting ART	None	Scale-up to 10% by 2011 [43]		
**ART if HIV/TB**	2010 level	None			
	Scale-up period		2011–2014	2011–2013	2011
	Final level [2,44,45]		100%	85%	66%
**ART**	PLHIV	30% [18]			
	PLHIV with CD4 < 350		Scale-up to 80% by 2012 [31]		
**Default**	Level of	8% [42]	1.5%	1.5%	8%

**Fig 2:**
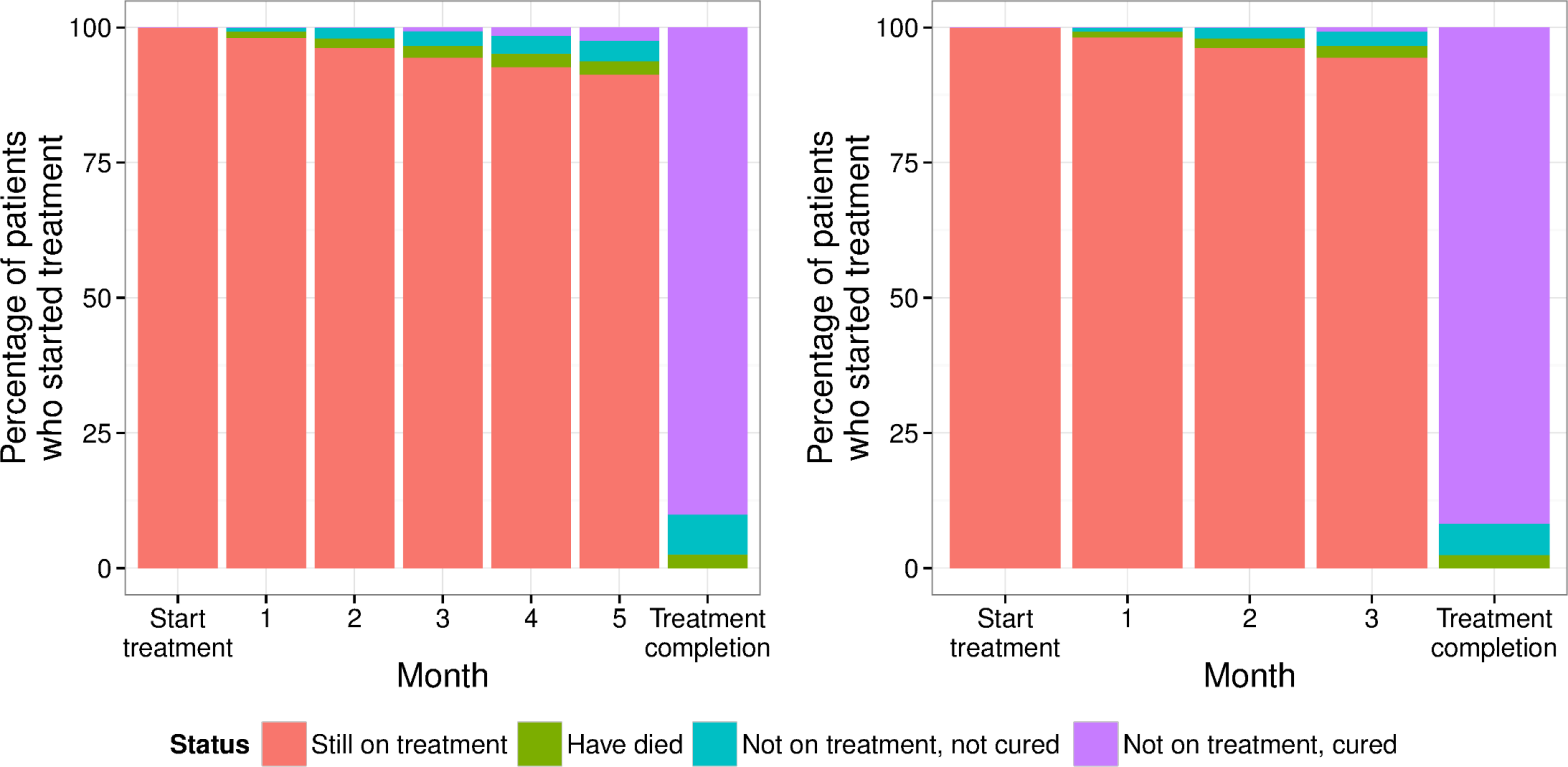
Assumed treatment outcomes at the end of each month for the standard 6-month TB regimen (left) vs. a new 4-month regimen (right) for a new TB patient without HIV. The cumulative percentage of the initial population starting treatment is shown with each of the different possible outcomes.

Individuals become non-infectious upon appropriate treatment initiation [20,21]. At each month of treatment, a proportion of individuals on treatment were lost to follow-up (with or without cure) and a further proportion die (Fig 2). These outcomes depend on HIV and ART status, resistance and whether an individual has received TB treatment before (Table 2).

**Table 2:** Treatment specific parameter values. * /_H_ = parameter does not differ by HIV status. ** /_R_ = parameter does not differ by resistance status. MDR = multi-drug resistance, resistance to standard regimen. ^a^Split in the ratio (3,2,1,1) over the first 4 months ^b^Both standard and new regimens are ineffective against MDR. Background rate of self-cure included.

Category	Parameter	HIV status*	Resistance**	Value [range]	Ref.
Initial resistance levels	Resistance percentages among new patients	/_H_	S	98.2%	[2]
		/_H_	MDR	1.8%	[2]
Outcomes(1^st^ line treatment)	Probability of mortality	Negative	S	0.025 [0.020–0.030] ^a^	[22]
		Negative	MDR	0.107 [0.080–0.130] ^a^	[22]
		Positive, not on ART	S	0.330 [0.330–0.430] ^a^	[26]
			MDR	0.850 [0.720–0.980] ^a^	[27]
		Positive on ART	S	0.070 [0.050–0.090] ^a^	[26]
			MDR	0.110 [0.107–0.210] ^a^	[27]
	Probability cure at treatment completion	/_H_	S	0.970 [0.950–0.980] ^b^	[40]
		/_H_	MDR	0.500 [0.400–0.550] ^b^	[22]
	Proportion who complete 3^rd^ month who are cured	/_H_	S	0.480 [0.300–0.600] ^b^	[23]
		/_H_	MDR	0.180 [0.110–0.250] ^b^	[23]
	Proportion who complete 4^th^ month who are cured	/_H_	S	0.660 [0.500–0.690] ^b^	[24,25]
		/_H_	MDR	0.280 [0.090–0.310] ^b^	[24,25]
	Percentage of patients who default	/_H_	/_R_	Differs by baseline	
Outcomes(Re-treatment)	Probability of mortality	Negative OR positive on ART	S	0.056 [0.040–0.070]	[22]
			MDR	0.145 [0.100–0.200]	[22]
		Positive, not on ART	S	0.167 [0.120–0.210]	[26,28,29]
			MDR	0.435 [0.300–0.600]	[26,28,29]
	Resistance percentages among re-treatment patients	/_H_	S	93.3% [89.8%-94.6%]	[2]
		/_H_	MDR	6.7% [5.4%-8.2%]	[2]

There are 18 separate treatment outcome combinations that depend on treatment regimen (standard, shortened or MDR), type (first or re-treatment) and HIV status (HIV+ no ART, HIV+ on ART, HIV-). For example, for new patients without HIV the treatment outcomes are given in Fig 2. These are composed of weighted averages over the resistance profiles (which differ if on re-treatment). Resistance (both MDR and to the new regimen) increases the probability of mortality [22] and decreases the probability of cure during and at treatment completion [23–25]. Those with HIV have a higher mortality, which is partially mitigated by ART [26], and is increased by TB resistance [27]. Outcomes during retreatment are worse, with higher mortality rates, which are also dependent on HIV and resistance status [22,26,28,29]. For further details on all parameters see Table B and the S1 Appendix.

### Model calibration

The model was calibrated using a Nelder-Mead simplex algorithm to minimize a sum of squares error term measuring the distance between TB incidence, HIV and ART data and the estimates by WHO [30], UNAIDS [31], and South African component of ZAMSTAR [32] (for more information see S1 Appendix). The model fitting used the base case parameters (see Table 1) except for the scenario of high default. The model was fitted separately to the data assuming this level of default.

### Intervention description

The new 4-month regimen was assumed to be non-inferior to the standard regimen, based on the criteria likely to be used to trial new regimens. This is also a conservative assumption for impact of a shortened regimen and was used to explore the impact of treatment shortening only. We did not include non-inferiority margins and instead set the two regimens to be equivalent in their outcomes. To match the treatment outcomes, it was assumed that no mortality occurs in the final 2 months of the standard regimen, that the same proportion of patients starting treatment were cured at either 6 months (standard) or 4 months (new), and that the proportion of those that default from treatment per month was the same. We assumed that at least two months treatment was required for any level of cure and that 3 months of the shortened regimen cured a greater proportion of the defaulters at 3 months than 3 months of the 6 month regimen (23% vs. 20%). The difference between the regimens is then that there are fewer defaults (due to those defaulting in months 5 and 6 of the standard regimen) and more of the early defaulters from the 4-month regimen are cured than from the standard regimen. This non-inferiority assumption results in relatively similar parameterization of outcomes during treatment (e.g. Fig 2).

The transmission model was calibrated across the 2004–2010 period. This is the base case scenario that then diverges into three baselines from 2011 to 2035. These three baselines are included to account for different analytical viewpoints–what would be the impact of a shortened regimen if guidelines were adhered to perfectly, or if we achieve our policy aims or if we continue at current levels of adherence? The three baselines are: “Guidelines” (assuming all existing TB and TB/HIV treatment guidelines are adhered to at 100%), “Policy” (assuming policy level coverage targets are achieved for TB and TB/HIV treatment where available or optimistic levels are assumed [1,33]) and “Current” (assuming maintenance of most recently measured coverage). The details of these baselines are given in Table 1, where it can be seen that TB control will be best in “Guidelines”, then “Policy”, then “Current”. The reference baseline was the “Policy” baseline.

The shortened 4-month regimen was introduced in 2015 with a rapid scale-up (see Fig B in S1 Appendix). This scale-up means that after 3 years 70% would receive the new regimen, after 5 years 80% and by 2025 under 10% of patients would continue to receive standard treatment. The impact of the shortened 4-month regimen is compared against continuation of use of the standard regimen in each of the three baselines across the 2015–2035 period. 2035 was chosen as the end date as it is a key deadline in the WHO Global Stop TB strategy [34].

### Scenario analysis

We focused on exploring scenarios that would have a large impact on our results, instead of investigating the small effects with a wider probabilistic sensitivity analysis that would have been computationally expensive with this type of model. Alongside our three baselines, a range of key scenario analyses was explored in the reference “Policy” baseline. Previous work has shown that levels of default drive much of the impact of shortened regimens [11,12]. We considered three levels of default: 1.5% (in “Guideline” and “Policy” baselines), 8% (in “Current” baseline) and 30% in a high default scenario analysis. Background levels of resistance could also affect regimen efficacy. Hence we also considered the impact of a high initial resistance level of 20% MDR in treatment-naïve patients. A further scenario matches previous modeling analysis [10] by assuming that treatment must be completed in order for cure to be achieved.

A separate analysis explored our assumptions around resistance dynamics. Instead of assuming constant levels, we considered the impact of a shortened regimen if MDR-TB levels quadrupled, by linear increase, in treatment-naïve patients by 2035.

### Costs

Costs were included from local patient and health system surveys (Table 3, [6,7,14]). Due to differences in adherence to TB treatment guidelines and therefore service utilization, the “Current” baseline had lower levels of patient and health system costs than the other two (“Guidelines” and “Policy”). Future values were discounted at a rate of 3% per year. Diagnostic costs were included for those with and without TB, assuming that an additional pool of possible TB patients were screened other than those on treatment with different coverage of GXP by different scenarios (see S1 Appendix). ART costs were also sourced from local patient and health system surveys [35,36].

**Table 3:** Healthcare provider and patient costs for all three baselines.

Category (length)	Split	Healthcare provider costs, mean [range]		Ref.	Patient costs, mean [range]		Ref.
		Guidelines / Policy	Current		Guidelines / Policy	Current	
First-line treatment (1mo, excl. drugs)	IP	200 [152–230.8]	60.5 [39.4–95.8]	[6]	148.7 [86.7–163.5]	59.73 [34.84–65.70]	[7]
	CP	53.9 [41.0–62.2]	16.3 [10.7–25.8]	[6]	116.8 [33.6–128.5]	27.38 [7.87–30.11]	[7]
MDR treatment (all)		10214.7 [8618.6–24579.6]		[6]	3318.5 [2986.6–3650.3]		[7]
ART (per year)		639.1 [575.2–703]		[35,36]	84.9 [76.5–93.4]		[7]
Diagnostics	Treatment of bacterial infection	17.6 [12.9–20.6]		[6]	23.7 [21.3–26]		[7]
	GeneXpert test	21.6 [14.6–28.4]		[6]	7.8 [7.0–8.6]		[7]
	Smear test	7.9 [5.1–10.6]		[6]	7.8 [7.0–8.6]		[7]
	Culture and DST	53.9 [38–69.8]		[6]	7.8 [7.0–8.6]		[7]
	X-Ray	24.1 [21.7–26.5]		[6]	3.9 [3.5–4.3]		[7]

The number of Disability Adjusted Life Years (DALYs) accumulated in each scenario was calculated using the standard formula with age weighting, with disability weights for TB cases that are HIV positive of 0.399 and for HIV negative 0.331 [37]. Using random sampling over a uniform distribution of the costs, the cost at which the cost per DALY averted, i.e. the incremental cost effectiveness ratio (ICER), was equal to a given willingness to pay (WTP) threshold from a societal perspective was calculated. This threshold cost includes the (here unknown) price of the drugs used within the regimen, plus the cost of other operational changes such as drug susceptibility tests required in order for the regimen to be used. The range reported is then linked to uncertainty in costs only. The time frame for the DALYs was 2015–2035. Three WTP thresholds were used, a quarter, a half and the 2015 full Gross Domestic Product (GDP) per capita in South Africa ($6,618) [38,39] (see S1 Appendix for details of this calculation). These calculations were performed with and without ART costs.

### Outcomes

Our primary outcome was the percentage of TB disease burden, deaths and DALYs averted by replacement of the standard first-line treatment with a shortened non-inferior 4-month regimen. Here TB burden was defined as the number of person years with TB disease. The price of the shortened regimens is currently unknown. Hence we focus our economic analysis on the threshold cost at which the regimen would be cost-effective at three WTP thresholds (1 x, ½ x & ¼ x GDP).

## Results

### Epidemiological and cost impact

The model predicts that, with a “Current” baseline and continued use of the standard regimen, there could be 6.4 million person years with TB disease and 2.1 million deaths in South Africa between 2015 & 2035 (Table 4). Using a non-inferior 4-month regimen in South Africa results in a 1% reduction in this TB burden in the “Current” baseline (Table 4). With a “Policy” or “Guideline” baseline, the impact was negligible (Table 4, Figs C & D in S1 Appendix).

**Table 4:** Results table. Epidemiological impact is estimated from the total estimated TB burden across 2015–2035 for predictions using the standard 6-month regimen (Std.) versus introduction of a new 4-month regimen (New) with no discounting. Also given is the percentage change from predictions using the standard regimen to use of the new regimen. The cost impact is given in terms of the patient (Pat.) costs averted (non-discounted) and the cost-effective 4-month regimen cost at three willingness-to-pay thresholds (multiples of GDP) using discounted values. The range reflects uncertainty in the costs. NA represents an invalid cost-effective cost not determined due to a lack of difference in impact or to it being negative. * This has units of person years with TB disease.

		Epidemiological impact						Cost-effectiveness			
*Baseline*	Regimen	Total TB case burden* (mill.)	Total deaths (mill.)	Total DALYs (mill.)	% difference relative to Std.			Total patient costs (bil.) (Mean[range])	Cost-effective new regimen cost(Mean [range])		
									WTP (GDP ($6,618))		
					Case burden	Death	DALY		1	½	¼
**Current**	Std.	6.4	2.1	34.9	/	/	/	0.4 [0.2, 0.5]	/	/	/
	New	6.3	2.1	34.5	-1	-1	-1	0.3 [0.2, 0.5]	436 [NA, 5983]	275 [NA, 3033]	194 [NA, 1559]
**Policy**	Std.	6.1	2.0	32.2	/	/	/	1.1 [0.6, 1.7]	/	/	/
	New	6.1	2.0	32.1	0	0	0	0.9 [0.5 1.3]	NA	NA	NA
**Guidelines**	Std.	6.2	1.8	28.6	/	/	/	1.0 [0.5,1.5]	/	/	/
	New	6.1	1.8	28.5	0	0	0	0.8 [0.5, 1.1]	NA	NA	NA
***Scenario analysis (Policy baseline)*:**											
**High default**	Std.	10.0	2.9	47.6	/	/	/	1.1 [0.8, 2.4]	/	/	/
	New	9.8	2.9	46.5	-2	-2	-2	0.9 [0.8, 1.8]	978 [NA, 5919]	639 [NA, 3131]	470 [NA, 1798]
**High resistance**	Std.	6.4	2.2	35.5	/	/	/	1.6 [1.3, 2.4]	/	/	/
	New	6.4	2.2	35.5	0	0	0	1.3 [1.2, 2.1]	NA	NA	NA
**Cure only at treatment completion**	Std.	6.6	2.1	34.6	/	/	/	1.1 [0.7, 1.8]	/	/	/
	New	6.4	2.1	33.8	-3	-2	-2	0.9 [0.6, 1.3]	991 [NA, 9300]	647 [NA, 4918]	475 [NA, 2728]
**Increasing MDR (1.8% to 7.2%)**	Std.	6.3	2.1	33.7	/	/	/	1.8 [0.8, 1.9]	/	/	/
	New	6.2	2.0	33.4	-1	-1	-1	1.6 [0.8, 1.6]	622 [NA, 7493]	613 [NA, 3764]	459 [NA, 2103]

The impact on patient costs due to the introduction of a shortened 4-month regimen was estimated to be more substantial (Table 4). Up to 20% of total patient costs could be averted in each of the three baselines.

Comparing across the three baselines (Table 4), it can be seen that the biggest TB burden was estimated to be in the “Current” baseline, with a lower burden in the “Policy” and then “Guidelines” baseline, as would be expected by the level of TB control. The epidemiological impact of the shortened regimen is also greatest in the “Current” baseline, compared to a negligible impact in the other two baselines.

### New regimen cost threshold for cost-effectiveness

The average monthly cost for the 4-month regimen at which the cost per DALY averted was equal to South Africa’s 2015 GDP per capita ($6,618) was $436 [NA, 5983] (mean [range]) for the “Current” baseline (Table 4). This results in a mean cost of approximately $14 per day. At costs below this threshold the regimen would be cost-effective and even cost-saving. A cost-effective regimen cost could not be calculated for the other two baselines as the difference in the TB burden averted was not sufficient.

As the willingness to pay (WTP) threshold decreases the maximum cost at which the 4-month regimen would be cost-effective decreases (Table 4). When the costs of antiretroviral therapy (ART) are included, the regimen cost decreases slightly (Table D in S1 Appendix). The differences in total patient costs when ART costs are included are negligible (Table E in S1 Appendix).

### Scenario analysis

The impact of the 4-month regimen remained similar in all scenarios explored, with no scenario causing the impact to increase to more than 3% (Table 4). The largest rises in impact were due to increases in the level of default and assuming that there was no cure until treatment completion (Table 4). If default increased to 30% in the policy baseline the shortened regimen could prevent 2% of person years with TB disease and the cost-effective cost would increase to $978 [NA, 5919] (Table 4). Similarly, if it was assumed that there was no cure until treatment completion then 3% of the TB burden could be averted with a predicted cost-effective cost of $991 [NA, 9300] (Table 4).

If the initial level of MDR-TB in treatment-naïve individuals increased to 20% in a policy baseline, then the impact of the shortened regimen increased marginally to 1% with a new cost-effective cost of $622 [NA, 7493] (Table 4). However, increasing the level of MDR-TB in treatment-naïve individuals over time resulted in no change in impact compared to assuming a constant level (Table 4).

## Discussion

Using a detailed mathematical model fitted to the TB epidemic in South Africa, we found that introducing a 4-month first-line TB regimen would have a modest impact on the number of person years with TB disease and number of TB deaths over a 20-year period. However due to the patient costs averted, it is likely that such a regimen could be highly cost-effective.

Our results suggest that the epidemiological impact of the introduction of a 4-month regimen would be approximately 1%. In general, this is due to the low indirect impact on *M*. *tuberculosis* transmission: those who start TB treatment become non-infectious within 2 weeks of treatment [20,21] and hence the majority of transmission is occurring from those outside of the cohort starting treatment. We assumed that the regimens were equivalent, in that the same proportion completing treatment were cured, that the similar relative proportions were cured over time (e.g. percentage cured at completion of 75% of regimen was the same), but also that the same proportion of cases defaulted from treatment each month. Thus the epidemiological impact of the shortened regimen is linked to the proportion of cases defaulting from treatment in months 5 and 6 of the standard treatment that were not cured, that would most likely have been cured by the shortened regimen (as they would have completed 4 months of treatment). In our analysis, as has been seen in previous models [11,12], varying this level of default did alter this impact, but here only marginally. This, and our other scenario analysis, suggests that this impact, albeit modest, of regimen shortening is robust.

A shortened regimen could potentially have a large impact on patient costs due to the reduction in time spent attending health clinics and taking treatment. These costs drive our results that a 4-month regimen is likely to be cost-effective from a societal perspective. The provider cost savings however are likely to be relatively small compared to patient cost savings and hence policy makers in South Africa may further wish to consider affordability. This is because these patient savings cannot be converted directly into resources to fund health care and the purchase of drugs, and the two months treatment shortening is from the continuous phase, when healthcare provider costs are relatively low. The threshold costs that we found should also be seen as relevant for drug pricing, but if necessary should include the costs of new diagnostic tests for resistance that are likely to accompany the introduction of a new regimen. Some treatment shortening regimens may include compounds new to TB treatment and thus not in the current diagnostic repertoire for resistant TB and these costs would be incremental as part of regimen introduction. With the inclusion of ART costs, the total costs increase dramatically and mask the slight differences in TB costs due to introduction of the 4-month regimen.

Comparing results between baselines it can be seen that adherence to TB and HIV treatment guidelines affects the epidemiological impact by pushing it to negligible with small levels of default (for “Policy” and “Guideline”). With higher adherence to guidelines however, a shorter regimen has a bigger impact on patient costs averted. Note that both the health system and patient costs are lower in the “Current” baseline. This suggests that a shortened regimen would have less of an epidemiological impact in a setting with existing high levels of TB control, but could avert a significant portion (~20%) of the patient cost burden.

Previous modelling work has estimated the impact of shortened regimens, although, to our knowledge, not with this specific linking to a setting. Our estimates were lower than previous modeling results [10–12] for several reasons. Firstly, we assumed that partial treatment can result in cure. This will act to decrease the impact of averting default via shortening treatment. In our scenario analysis we assumed that cure can only occur at treatment completion, as in [10] and found an increased impact of introduction of the shortened regimen. Secondly, we assumed that the shortened regimen was non-inferior, based on the current trial designs currently being used for new TB drugs. Hence the same proportion of patients starting treatment died during treatment, as opposed to previous work [11,12]. Thirdly, we considered a time period for impact of 20 years (2015–2035) (shorter than [10]) across which continuation of TB control measures meant that incidence was declining and hence potential impact would decrease over time as opposed to assuming constant equilibrium incidence [12]. Fourthly, we assumed a scale-up of introduction of the 4-month regimen resulting in a delayed, more realistic impact than straight replacement as first-line therapy. As our scale-up was relatively rapid, it is unlikely that a shorter scale-up that still captures operational realities (i.e. not a straight replacement), would significantly alter our results.

The limitations of our modeling relate to uncertainty–both in our limited analysis of it and in future levels of control and resistance. However, our scenario analyses revealed the results, around the small impact of a regimen shortening, to be robust. Specifically, we did not include the most recent South African ART initiation guidelines–hence we may over predict levels of HIV and hence TB disease in the future. However, as we considered the relative impact of changing TB treatment and our cost-effectiveness results are driven by TB treatment costs not by ART costs (Table E in S1 Appendix), we would not expect this to have a large impact on our results. A limitation of our model structure was that whilst rates of developing TB in those with HIV were modeled as depending on CD4 count, characteristics of TB disease, including typical duration and smear positive rates, depended on HIV status but not level of immune status (i.e. CD4 count). The limitations of our cost-effectiveness analysis are linked to uncertainty in future costs and prices. We thus adopted a threshold analysis. We also did not consider the continuation of indirect effects past our 2035 deadline and did not include regimen development costs, or the economic effects of increasing resistance. It should however, be noted that the extensive use of primary recent cost data is a strength of this paper.

The results of this work suggest that for policymakers adopting a new 4-month regimen would be an effective strategy due to the patient cost savings. We here investigated only the impact of regimen shortening, with the conservative assumption of non-inferiority. Any regimen shown to be superior, which would require a different trial design than has currently been utilized, would have a larger impact on TB burden. Similarly, if we had included margins of non-inferiority, i.e. allowed the shortened regimen to be better or worse within some range that still allowed it to be defined as “non-inferior”, the impact on TB burden would increase or decrease respectively. Future work could explore this threshold for cost-effectiveness, although our results suggest that even a slightly inferior regimen could remain cost-effective due to the patient savings from shortened treatment.

In conclusion, it is unlikely that a new shortened 4-month regimen would stop the spread of TB in South Africa with only a small impact on ongoing transmission. However, the costs averted for those with TB that are detected and enrolled on TB treatment would be large. These averted costs, which are dependent on levels of adherence to TB and HIV treatment guidelines, mean that new 4-month regimens are likely to be highly cost-effective in South Africa.

## Supporting Information

S1 Appendix Supporting information on model structure and additional results. (DOCX)
