# Supplementary material for: The Impact and Cost-Effectiveness of a Four-Month Regimen for First-Line Treatment of Active Tuberculosis in South Africa
Source: PLoS One. 2015 Dec 30;10(12):e0145796. doi: 10.1371/journal.pone.0145796 (PMC4696677; doi:10.1371/journal.pone.0145796)
Supplement: S1 Appendix — (DOCX) [file pone.0145796.s001.docx]

**Supplementary for**

**“The impact and cost-effectiveness of a four-month regimen for first-line treatment of active tuberculosis in South Africa”**

Gwenan M Knight, Gabriela B Gomez, Peter J Dodd, David Dowdy, Alice Zwerling, William A Wells, Frank Cobelens, Anna Vassall, Richard G White

**Contents**

***1) Model and parameterisation***

*Model details (with parameter table)*

*Model calibration*

*New regimen scale-up*

*MDR treatment outcomes*

***2) Cost calculations***

*Diagnostic costs*

*Cost-effective regimen price*

***3) Additional results***

*Impact on TB burden*

*Impact on new regimen cost-effective price with ART costs included*

*Impact on patient costs with ART costs included*

***References***

***1) Model and parameterisation***

***Model details***

The individual-based simulation model was built using C and has been previously published [1,2]. All parameters are found in Table A.

*Natural history of TB*

The TB model structure follows along the lines of the simplified representation in Figure 1. Individuals are born without *M.tb* infection; can acquire an *M.tb* infection which may, or may not develop into active TB disease; and individuals may, or may not have their active TB disease detected and initiate treatment. The force-of-infection for TB is proportional to the prevalence of active TB, weighted by the infectiousness of each prevalent TB case. We assume age-dependent mixing between different age groups, parameterized with data from a social contact study carried out in South Africa as part of ZAMSTAR [3].

*TB in HIV-uninfected individuals*

Upon infection with *M.tb*, individuals’ time-since-infection is tracked and they are subject to a hazard of developing pulmonary TB disease per unit time. This hazard is higher in the first 2 years following (re-)infection, with an age-dependence following that used in [4]. In subsequent years, the hazard of activation assumes a lower constant value. Individuals with an *M.tb* infection have a partial protection against reinfection [5], however, a successful re-infection is treated identically to an initial infection.

Upon activation to pulmonary TB, 65% of cases in 20 year olds were assumed smear positive among those without HIV infection, following the age pattern reported in [4], smear negative TB assumed 23% as infectious as smear positive TB [6,7]. Active TB without treatment results in death (70% for smear-positive disease; 30% for smear-negative disease) or else self-cure over a timescale of 3 years based on [8]. These alternatives are modelled as proportional hazards with a Weibull-distributed time-to-event. Self-cure leaves individuals with an *M.tb* infection, and acts like a new infection in terms of activation risks.

Detection and initiation of treatment in those with active TB disease is assumed to occur with a probability taken as the WHO estimate of the case-detection rate [9]. The timing of detection and treatment initiation occurs at a certain fraction of an individual’s time-to-outcome without detection and treatment (i.e. more rapidly progressing TB disease is detected proportionately more rapidly).

**HIV**

*HIV infection and life-expectancy*

HIV incidence through calendar time was taken from a deterministic model of HIV transmission [10]. HIV infections are distributed by age according to a Weibull distribution with shape parameter k=2.3 and scale s=25.9 years matching the gender-average of data presented in [11]. Life expectancy for those infected by HIV without ART is modelled by a Weibull distribution with parameters (k=2.3, s=13.3 years), from a weighted least-squares fit to the survival data from the CASCADE collaboration [12].

*HIV & TB*

The influence of HIV on the risks of developing TB is mediated through CD4 count, which is modelled continuously for each individual. The CD4 cell count trend and incidence rate ratio for developing TB in HIV-infected individuals not receiving ART are modelled similarly to [13]. Upon infection, CD4 cell count drops by 25% from an initial value of 1000 cells per microliter, followed by a linear decline to zero at death. Individuals’ TB incidence rate ratio (IRR) increases exponentially with decreasing CD4 cell count with a rate 0.36 per 100 cells per microliter. This IRR applies to the hazard of an *M.tb* infection becoming active TB disease as modelled above. HIV-infected individuals are assumed not to have any protection from a prior *M.tb* infection against reinfection disease.

HIV-infected active TB cases were taken to be 45% as likely to be smear positive as HIV-uninfected TB cases [13]. Untreated active TB disease in HIV-infected individuals was assumed to have 100% case-fatality, over a time-scale of 5 months [14], also modelled as a Weibull distribution. The probability of detection and treatment initiation, and its timing, was modelled as for TB in HIV-uninfected individuals.

*ART & ART coverage*

The life expectancy of an individual starting ART was modelled as in [15]: for someone of age *a* initiating ART at CD4 count *CD4_A_* whose CD4 count immediately following seroconversion was *CD4_1_*, their life-expectancy is taken as:

$$max({LE}_{0}-a,0)\times\left( \frac{{CD4}_{A}}{{CD4}_{1}} \right)^{0.5}$$

where *LE_0_* is the individual’s life-expectancy in the absence of HIV infection.

The effect of ART on TB incidence was taken to be equivalent to returning CD4 count to that following seroconversion, resulting in a population IRR of approximately 0.3 for TB in those on ART compared with HIV-infected individuals not on ART.

ART initiation was modelled as for the PopART model used in [16]. Briefly, individuals who were classified as in contact with health services and whose CD4 count was below the threshold of 350 cells per microliter were assumed to commence ART at a rate of 2 per year. The rate of becoming in contact with health services was modelled by a function of the form:

$$A_{max}\times\frac{\left[ \left( t-t_{0} \right)/s \right]^{k}}{1+\left[ \left( t-t_{0} \right)/s \right]^{k}}$$

where *t_0_* was taken as 2004, and the other parameters were fitted to reported South African ART coverage. In this work we kept the same shape for this function, (*s*,*k*) = (15.2 *years*, 1.9), but refitted the overall scale *A_max_* to the ART prevalence in HIV-infected individuals measured by the ZAMSTAR prevalence survey. Drop out from ART was taken as 5% per year. HIV-infected individuals with detected TB disease were initiated on ART with a specified coverage after 2010.

Individuals become non-infectious upon treatment initiation [17].

**Demography**

The model is populated in 1980 with 25,000 individuals whose characteristics are taken from the ZAMSTAR prevalence survey. The results are multiplied up to the full population of SA at the end of each simulation. The initial prevalence of active TB was based on the WHO estimate of TB prevalence for 1990 and the number on treatment implied by the case-detection ratio. The appropriate numbers of active TB cases and individuals on TB treatment were selected randomly from amongst adults. The prevalence of latent *M.tb* infection was determined by assuming that the force-of-infection generated by this prevalence had been constant for long enough to achieve equilibrium. *M.tb* infections were distributed by age as for an equilibrium, as were the times since *M.tb* infection. These were the initial conditions for the model. The model was then simulated until 2010 and the output compared to the WHO data (see “Model calibration” below). If the output did not match the data then certain parameters were altered until the model matched the data.

Age-specific mortality was implemented using the 1-parameter UN life-tables, with the calendar time dependence via the UN ESA estimates of life-expectancy of birth for each year [18], with the HIV-induced dip smoothed out. The UN ESA estimates of crude birth rate and in- and out-migration were also used, with immigrating individuals randomly cloning adults from the population. Finally, if necessary, a small correction was applied to birth and death rates if the relative population size over- or under-shot the UN ESA population growth prediction for South Africa, to ensure a matching trend.

| **parameter** | **value** | **reference** |
| --- | --- | --- |
| Smear positive transmission coefficient, beta | 14.5/y | Fitted |
| Endogenous reactivation (%/y) | 0.7%/y | Fitted |
| Probability of progression <2 years after infection | 13.4% | [4] |
| Protection from latent *M.tb* | 70% | Fitted |
| Fractional timing of detection | 20% | Fitted |
| Smear-positivity in 20 year olds (HIV-) (%) | 65% | [4] |
| Relative infectiousness of smear-negative TB | 23% | [6,7] |
| Case fatality in untreated smear-positive TB (HIV-) | 70% | [8] |
| Case fatality in untreated smear-negative TB (HIV-) | 30% | [8] |
| Duration of untreated TB disease (HIV-) | Weibull(1.5, 3.25y) | [8] |
| Probability of detection & treatment for a TB case | 72% | [9] |
| log(IRR for TB) / CD4 decrement | 0.36/(100 cells/μL) | [13] |
| Risk-ratio for smear-positivity (HIV+) | 45% | [19] |
| Case fatality in untreated TB (HIV+) | 100% | [14] |
| Relative duration of untreated TB disease (HIV+/HIV-) | 10% | [14] |
| HIV life-expectancy without ART | Weibull(2.3,13.3y) | [12] |
| HIV incidence age pattern | Weibull(2.3,25.9y) | [11] |
| HIV incidence peak | 2%/y | Fitted |
| HIV incidence peak year | 1997 | Fitted |
| HIV incidence peakiness | 20 | Fitted |
| HIV incidence theta | 3.2 | Fitted |
| ART hazard (*k,s*) | (1.9,15.2 y) | [16] |
| ART hazard scale, *A_max_* | 0.6 | Fitted |
| Birth rates and background mortality | NA | [18] |

Table A: Natural history parameters used in the model in the Basecase with default at 8%.

***Model calibration***

The Nelder-Mead simplex algorithm used an error term that was constructed by rescaling each time-series to be comparable and additionally scaling the contribution at each time point by the inverse of the confidence interval. The total error was the sum of SSEs of this type for TB prevalence, TB incidence, HIV-TB incidence, TB incidence in 2010, HIV prevalence and ART coverage in all those with HIV infections in 2010 [20]. The parameters varied to achieve a fit were the transmission coefficient (beta), the endogenous reactivation rate, the protection from latent *M.tb* infection, the timing of detection as a fraction of time to disease resolution, the overall scale to the ART coverage hazard, and the peak HIV incidence. The other parameters governing the shape of the HIV incidence were obtained from an analogous fit to HIV prevalence alone.

The fit of the model to the data is shown in Figure A.

Figure A: Model fit to data: this is the average model incidence output all forms and in HIV positives across the 2004-2010 period (*) plotted against the data [20].

***New regimen scale-up***

A new regimen would not replace the standard regimen immediately. Instead, the new 4-month regimen was introduced gradually after 2015 (Figure B). Hence the comparison is between the TB burden with 100% use of the standard regimen (HRZE) for first line therapy vs. increasing use of the 4-month regimen and some use of the standard regimen (Figure B).

Figure B: Proposed coverage of the new shortened regimen by end of year with an introduction in 2015.

***MDR Treatment outcomes***

The MDR treatment outcomes are given as overall parameters in Table B and as individual monthly parameters in Table C.

| **Category** | **Parameter** | **HIV status*** | **Resistance**** | **Value [range]** | **Notes** | **Ref.** |
| --- | --- | --- | --- | --- | --- | --- |
| Outcomes  (MDR treatment) | Probability of mortality | N | MDR | 0.160 [0.140-0.180] | Split in the ratio (3,2,1,1) over the first 4 months | [21] |
|  |  | P, no ART | MDR | 0.300 [0.250-0.350] |  |  |
|  |  | P, on ART | MDR | 0.220 [0.180-0.250] |  |  |
|  | Probability of default | /_H_ | MDR | 0.220 [0.155-0.285] | 50% default in the first 4 months, few default after 10 months [22]. | [21,23] |
|  | Probability cure if default | /_H_ | MDR | Based on 1^st^ line parameterisation | 10% more cured in the first 6 months than 1^st^ line. Linear proportion cured to 80% of those that default at month 24. | Assumed |

Table B: Treatment specific parameter values. * /­­_H_ = parameter does not differ by HIV status. N = HIV negative, P = HIV positive, ART = antiretroviral therapy. ** /_R_ = parameter does not differ by resistance status. MDR = multi-drug resistance, resistance to standard regimen. newR = resistance to new regimen.

|  | **Default** | | | **Proportion cured** | | | **Proportion who default and are cured** | | |
| --- | --- | --- | --- | --- | --- | --- | --- | --- | --- |
|  | **Mean** | **Low** | **High** | **Mean** | **Low** | **High** | **Mean** | **Low** | **High** |
| **1** | 0.010 | 0.008 | 0.012 | 0.000 | 0.000 | 0.000 | 0.000 | 0.000 | 0.000 |
| **2** | 0.015 | 0.012 | 0.018 | 0.000 | 0.000 | 0.000 | 0.000 | 0.000 | 0.000 |
| **3** | 0.025 | 0.020 | 0.030 | 0.480 | 0.400 | 0.560 | 0.012 | 0.008 | 0.017 |
| **4** | 0.045 | 0.030 | 0.060 | 0.530 | 0.450 | 0.600 | 0.024 | 0.014 | 0.036 |
| **5** | 0.030 | 0.025 | 0.035 | 0.580 | 0.500 | 0.650 | 0.017 | 0.013 | 0.023 |
| **6** | 0.020 | 0.015 | 0.025 | 0.610 | 0.550 | 0.690 | 0.012 | 0.008 | 0.017 |
| **7** | 0.005 | 0.003 | 0.007 | 0.600 | 0.600 | 0.750 | 0.003 | 0.002 | 0.005 |
| **8** | 0.005 | 0.003 | 0.007 | 0.620 | 0.606 | 0.759 | 0.003 | 0.002 | 0.005 |
| **9** | 0.005 | 0.003 | 0.007 | 0.630 | 0.612 | 0.768 | 0.003 | 0.002 | 0.005 |
| **10** | 0.005 | 0.003 | 0.007 | 0.640 | 0.618 | 0.776 | 0.003 | 0.002 | 0.005 |
| **11** | 0.005 | 0.003 | 0.007 | 0.650 | 0.624 | 0.785 | 0.003 | 0.002 | 0.005 |
| **12** | 0.005 | 0.003 | 0.007 | 0.670 | 0.629 | 0.794 | 0.003 | 0.002 | 0.006 |
| **13** | 0.005 | 0.003 | 0.007 | 0.680 | 0.635 | 0.803 | 0.003 | 0.002 | 0.006 |
| **14** | 0.005 | 0.003 | 0.007 | 0.690 | 0.641 | 0.812 | 0.003 | 0.002 | 0.006 |
| **15** | 0.005 | 0.003 | 0.007 | 0.700 | 0.647 | 0.821 | 0.004 | 0.002 | 0.006 |
| **16** | 0.005 | 0.003 | 0.007 | 0.710 | 0.653 | 0.829 | 0.004 | 0.002 | 0.006 |
| **17** | 0.005 | 0.003 | 0.007 | 0.720 | 0.659 | 0.838 | 0.004 | 0.002 | 0.006 |
| **18** | 0.005 | 0.003 | 0.007 | 0.730 | 0.665 | 0.847 | 0.004 | 0.002 | 0.006 |
| **19** | 0.005 | 0.003 | 0.007 | 0.740 | 0.671 | 0.856 | 0.004 | 0.002 | 0.006 |
| **20** | 0.005 | 0.003 | 0.007 | 0.760 | 0.676 | 0.865 | 0.004 | 0.002 | 0.006 |
| **21** | 0.005 | 0.003 | 0.007 | 0.770 | 0.682 | 0.874 | 0.004 | 0.002 | 0.006 |
| **22** | 0.000 | 0.000 | 0.000 | 0.780 | 0.688 | 0.882 | 0.000 | 0.000 | 0.000 |
| **23** | 0.000 | 0.000 | 0.000 | 0.790 | 0.694 | 0.891 | 0.000 | 0.000 | 0.000 |
| **24** | 0.000 | 0.000 | 0.000 | 0.800 | 0.700 | 0.900 | 0.000 | 0.000 | 0.000 |
| **Total** | 0.220 | 0.155 | 0.285 | / | / | / | / | / | / |

Table C: Proportion of patients on MDR treatment who default, of those who default (or complete treatment) the proportion that are cured and the combination of the two i.e. the proportion who default that are cured per month.

***2) Cost calculations***

***Diagnostic costs***

To estimate the cost of diagnosis it was assumed that for every TB case started on treatment, there would be an additional 23% diagnosed. This accounts for a primary default value of 19% [24-26]. It was assumed that every diagnosed case was GeneXpert positive. In South Africa there were 9.5-11x more suspects than smear positives (electronic TB register South Africa) and that there are 1.35x as many GeneXpert positive as smear positive [27]. This implies that there are ~7.5x as many suspects than GeneXpert positives. It was assumed that 30% of all patients on treatment received antibiotics and an X-ray. For those patients that start MDR treatment it was assumed that they have also received a culture and DST result. The coverage of GeneXpert (GXP) varies by baseline.

Thus for every 100 patients on treatment in the model, 123 would have been diagnosed. All these would have been GeneXpert positive. The pool of suspects potentially tested with GeneXpert is 7.5x bigger at 923 patients. However, dependent on the coverage of GeneXpert in each scenario, the number tested with GeneXpert can be smaller than 923.

Let **T** be the number of patients starting any non-MDR treatment, **M** be the number of patients starting MDR treatment and **C** the coverage of GeneXpert in the relevant scenario. Then the total diagnostic costs are:

Diagnostic costs = cost of all GeneXpert tests

+ (cost of antibiotics+X-ray)

+ (cost of MDR diagnosis)

= **T** x 1.23 x 7.5 x **C** x (cost of GeneXpert)

+ **T** x 0.3 x [cost of antibiotics+X-ray]

+ **M** x (1.23 x 7.5 x **C** x (cost of GeneXpert) + costs of culture + DST)

At a higher false negative rate for GeneXpert the pool of suspects would increase and hence we would have higher diagnostic costs.

***Cost-effective regimen price***

Cost per DALY averted

= (Netcosts) / (# DALYs averted)

= (Difference in treatment costs standard vs. short.) / (# DALYs averted) (1)

The total treatment costs compromise of the costs associated with using the standard regimen, the shortened regimen and second line treatments, as well as diagnosis. Let **U**, **S** and **M** be the number of treatments of standard (usual), shortened and second line treatments respectively with costs of c_U_, c_S_ and c_M_. The cost of diagnosis is **D**. Let subscript S denote costs incurred during roll out of the shortened regimen. (1) is then rearranged to (2). To be cost-effective, the cost per DALY averted must equal the GDP of South Africa and so (2) can be rearranged to (3).

Cost per DALY averted

=(U_S_ x c_U_ + S_S_ x c_S_ + M_S_ x c_M­_ + D_S_) - (U x c_U_ + M x c_M_ + D) (2)

(# DALYs averted)

GDP = (U_S_ x c_U_ + S_S_ x c_S_ + M_S_ x c_M­_ + D_S_) - (U x c_U_ + M x c_M_ + D)

(# DALYs averted)

GDP*(# DALYs averted) = (U_S_ x c_U_ + M_S_ x c_M­_ + D_S_) - (U x c_U_ + M x c_M_ + D) + S_S_ x c_S_

c_S_ = (U x c_U_ + M x c_M_ + D) – (U_S_ x c_U_ + M_S_ x c_M­_ + D_S_) + GDP*(# DALYs averted) (3)

S_S_

The cost effective price was also determined at a half and a quarter of the GDP to explore other potential willingness to pay thresholds. All values included were discounted at a rate of 3% a year.

***3) Additional results***

***Impact on TB burden***

The impact on the total TB burden was small (Table 4). This can be seen in the small divergence in incidence and mortality rates across the 2015-2035 period in the “Policy” baseline (Figure C&D).

Figure C: The epidemiological impact of a new shortened regimen on annual TB incidence is small. Shown here is an example of the output in the “Policy” baseline.

Figure D: The epidemiological impact of a new shortened regimen on annual TB mortality is small. Shown here is an example of the output in the “Policy” baseline.

***Impact on new cost-effective regimen price with ART costs included***

|  | **Regimen** | **Cost impact** | | |
| --- | --- | --- | --- | --- |
| ***Baseline*** |  | **Cost-effective new regimen price** | | |
|  |  | **WTP (GDP ($))** | | |
|  |  | **1** | **½** | **¼** |
| **Current** | Std. | / | / | / |
|  | New | 430  [NA, 5774] | 268  [NA, 2880] | 187  [NA, 1506] |
| **Policy** | Std. | / | / | / |
|  | New | NA | NA | NA |
| **Guidelines** | Std. | / | / | / |
|  | New | NA | NA | NA |
| ***Scenario analysis (Policy baseline):*** | | | | |
| **High default** | Std. | / | / | / |
|  | New | 964  [NA, 5750] | 625  [NA, 3002] | 456  [NA, 1707] |
| **High resistance** | Std. | / | / | / |
|  | New | NA | NA | NA |
| **Cure only at treatment completion** | Std. | / | / | / |
|  | New | 975  [NA, 7367] | 631  [NA, 3774] | 459  [NA, 2052] |
| **Increasing MDR resistance in treatment-naïve patients**  **(1.8% to 7.2%)** | Std. | / | / | / |
|  | New | 613  [NA, 9130] | 650  [NA, 4748] | 368  [NA,2558] |

Table D: Results table with ART costs. The cost-effective 4-month regimen price at three willingness-to-pay thresholds (multiples of GDP) is presented using discounted values. The range reflects uncertainty in the costs. NA represents an invalid cost-effective price not determined due to a lack of difference in impact or to it being negative.

***Impact on patient costs with ART costs included***

| **Baseline** | **Regimen** | **No ART** | **With ART** |
| --- | --- | --- | --- |
|  |  | **($ billions)** | **($ billions)** |
| **Current** | Std. | 0.4 [0.2, 0.5] | 6.5 [5.6, 7.4] |
|  | New | 0.3 [0.2, 0.5] | 6.5 [5.6, 7.4] |
| **Policy** | Std. | 1.1 [0.6, 1.7] | 6.3 [5.4, 7.2] |
|  | New | 0.9 [0.5 1.3] | 6.3 [5.4, 7.2] |
| **Guidelines** | Std. | 1.0 [0.5,1.5] | 5.6 [4.9, 6.5] |
|  | New | 0.8 [0.5, 1.1] | 5.6 [4.9, 6.6] |
| **Baseline: Policy, scenario:** | |  |  |
| **High default** | Std. | 1.1 [0.8, 2.4] | 7.6 [5.3, 8.7] |
|  | New | 0.9 [0.8, 1.8] | 7.7 [5.3, 8.7] |
| **High resistance** | Std. | 1.6 [1.3, 2.4] | 6.2 [5.3, 7.1] |
|  | New | 1.3 [1.2, 2.1] | 6.2 [5.3, 7.2] |
| **Cure only at treatment completion** | Std. | 1.1 [0.7, 1.8] | 6.2 [5.4, 7.2] |
|  | New | 0.9 [0.6, 1.3] | 6.2 [5.4, 7.2] |
| **Increasing MDR**  **(1.8% to 7.2%)** | Std. | 1.8 [0.8, 1.9] | 6.2 [5.3, 7.1] |
|  | New | 1.6 [0.8, 1.6] | 6.2 [5.3, 7.1] |

Table E: Total patient costs over 2015-2035 with discounting in the Policy baseline with use of the standard regimen or the new 4-month regimen.

References

1. Pretorius C, Menzies NA, Chindelevitch L, Cohen T, Cori A, et al. (2014) The potential effects of changing HIV treatment policy on tuberculosis outcomes in South Africa: results from three tuberculosis-HIV transmission models. AIDS 28 Suppl 1: S25-34.

2. Knight GM, Dodd PJ, Grant AD, Fielding KL, Churchyard GJ, et al. (2015) Tuberculosis prevention in South Africa. PLoS One.

3. Dodd PJ, Looker C, Plumb I, Bond G, Schaap A, et al. (In review) *Mycobacterium tuberculosis infection* incidence and social contact patterns in Zambia and South Africa. In: Unpublished, editor.

4. Vynnycky E, Fine PE (1997) The annual risk of infection with Mycobacterium tuberculosis in England and Wales since 1901. Int J Tuberc Lung Dis 1: 389-396.

5. Andrews JR, Noubary F, Walensky RP, Cerda R, Losina E, et al. (2012) Risk of progression to active tuberculosis following reinfection with *Mycobacterium tuberculosis*. Clin Infect Dis 54: 784-791.

6. Behr MA, Warren SA, Salamon H, Hopewell PC, Ponce de Leon A, et al. (1999) Transmission of *Mycobacterium tuberculosis* from patients smear-negative for acid-fast bacilli. Lancet 353: 444-449.

7. Tostmann A, Kik SV, Kalisvaart NA, Sebek MM, Verver S, et al. (2008) Tuberculosis transmission by patients with smear-negative pulmonary tuberculosis in a large cohort in the Netherlands. Clin Infect Dis 47: 1135-1142.

8. Tiemersma EW, van der Werf MJ, Borgdorff MW, Williams BG, Nagelkerke NJ (2011) Natural history of tuberculosis: duration and fatality of untreated pulmonary tuberculosis in HIV negative patients: a systematic review. PLoS One 6: e17601.

9. WHO (2013) TB data. Available: <http://www.who.int/tb/country/en/>. Accessed 2015 March 2

10. Fraser C, Cori A (2012) HIV model to investigate the PopART intervention.

11. Stover J, Johnson P, Hallett T, Marston M, Becquet R, et al. (2010) The Spectrum projection package: improvements in estimating incidence by age and sex, mother-to-child transmission, HIV progression in children and double orphans. Sex Transm Infect 86 Suppl 2: ii16-21.

12. Collaborative Group on AIDS Incubation and HIV Survival including the CASCADE EU Concerted Action C (2000) Time from HIV-1 seroconversion to AIDS and death before widespread use of highly-active antiretroviral therapy: a collaborative re-analysis. . Lancet 355: 1131-1137.

13. Williams BG, Granich R, De Cock KM, Glaziou P, Sharma A, et al. (2010) Antiretroviral therapy for tuberculosis control in nine African countries. Proc Natl Acad Sci U S A 107: 19485-19489.

14. Dimairo M, Mativenga S, Dauya E, Makamure B, Mangwanya D, et al. (2009) The fate of sputum smear-negative TB suspects managed by routine clinical services in Harare, Zimbabwe. CROI. Montreal, Canada.

15. Dodd PJ, Knight GM, Lawn SD, Corbett EL, White RG (2013) Predicting the long-term impact of antiretroviral therapy scale-up on population incidence of tuberculosis. PLoS One 8: e75466.

16. Eaton JW, Menzies NA, Stover J, Cambiano V, Chindelevitch L, et al. (2013) Health benefits, costs, and cost-effectiveness of earlier eligibility for adult antiretroviral therapy and expanded treatment coverage: a combined analysis of 12 mathematical models. Lancet Global Health.

17. Sepkowitz KA (1996) How contagious is tuberculosis? Clin Infect Dis 23: 954-962.

18. Department of Economics and Social Affairs PD (2011) World Population Prospects: The 2010 Revision. In: United Nations, editor. New York.

19. Getahun H, Harrington M, O'Brien R, Nunn P (2007) Diagnosis of smear-negative pulmonary tuberculosis in people with HIV infection or AIDS in resource-constrained settings: informing urgent policy changes. Lancet 369: 2042-2049.

20. WHO (2011) Global Tuberculosis Control.

21. Farley JE, Ram M, Pan W, Waldman S, Cassell GH, et al. (2011) Outcomes of multi-drug resistant tuberculosis (MDR-TB) among a cohort of South African patients with high HIV prevalence. PLoS One 6: e20436.

22. Jenkins HE, Ciobanu A, Plesca V, Crudu V, Galusca I, et al. (2013) Risk factors and timing of default from treatment for non-multidrug-resistant tuberculosis in Moldova. Int J Tuberc Lung Dis 17: 373-380.

23. Brust JC, Gandhi NR, Carrara H, Osburn G, Padayatchi N (2010) High treatment failure and default rates for patients with multidrug-resistant tuberculosis in KwaZulu-Natal, South Africa, 2000-2003. Int J Tuberc Lung Dis 14: 413-419.

24. Botha E, den Boon S, Lawrence KA, Reuter H, Verver S, et al. (2008) From suspect to patient: tuberculosis diagnosis and treatment initiation in health facilities in South Africa. Int J Tuberc Lung Dis 12: 936-941.

25. Botha E, Den Boon S, Verver S, Dunbar R, Lawrence KA, et al. (2008) Initial default from tuberculosis treatment: how often does it happen and what are the reasons? Int J Tuberc Lung Dis 12: 820-823.

26. Dunbar R, Lawrence K, Verver S, Enarson DA, Lombard C, et al. (2011) Accuracy and completeness of recording of confirmed tuberculosis in two South African communities. Int J Tuberc Lung Dis 15: 337-343.

27. Boehme CC, Nicol MP, Nabeta P, Michael JS, Gotuzzo E, et al. (2011) Feasibility, diagnostic accuracy, and effectiveness of decentralised use of the Xpert MTB/RIF test for diagnosis of tuberculosis and multidrug resistance: a multicentre implementation study. Lancet 377: 1495-1505.
